# Quantitative Change of Hepatitis B Surface Antigen Leading to Final Hepatitis B Surface Antigen Loss in Patients with Chronic Hepatitis B Receiving Nucleos(t)ide Analogs in China

**DOI:** 10.14309/ctg.0000000000000820

**Published:** 2025-01-16

**Authors:** Tianhui Zhou, Meng Shu, Fangyun Luo, Sijia Dong, Jiaming Teng, Yanan Du, Hong Qiu, Wei Cai

**Affiliations:** 1 Department of Infectious Diseases, Ruijin Hospital, Shanghai Jiao Tong University School of Medicine, Shanghai, China;; 2 Global Epidemiology, Office of the Chief Medical Officer, Johnson & Johnson, Shanghai, China;; 3 Hepatopathy Department, The Fifth People's Hospital of Ganzhou & Ganzhou Institute of Hepatology, Ganzhou, Jiangxi, China;; 4 Global Epidemiology, Office of the Chief Medical Officer, Johnson & Johnson, Titusville, New Jersey, USA.

**Keywords:** chronic hepatitis B, nucleos(t)ide analogs, qHBsAg, HBsAg loss

## Abstract

**INTRODUCTION:**

Loss of hepatitis B surface antigen (HBsAg) is the pivotal component of functional cure in patients suffering from chronic hepatitis B (CHB). The predictive value of quantitative HBsAg (qHBsAg) in HBsAg loss among those undergoing nucleos(t)ide analog (NAs) therapy is an area of ongoing investigation.

**METHODS:**

A retrospective cohort study using electronic medical records was performed. CHB patients with NAs treatment between January 1, 2012, and December 31, 2020 were enrolled and followed up until discontinuation of NAs, as indicated by a gap more than 12 months in prescription refills, past medical record, or study end. Patients were grouped into NAs treatment-naïve cohort and treatment-experienced cohort. In both cohorts, Cox regression models assessed associations between 12-month reduction in qHBsAg, baseline qHBsAg, and HBsAg loss.

**RESULTS:**

Overall, 2,627 CHB patients with NAs treatment was identified, including 1,179 in treatment-naïve cohort and 1,448 in treatment-experienced cohort. In treatment-naïve cohort, 9 patients had HBsAg loss (0.51/100 person-years). In treatment-experienced cohort, 30 patients had HBsAg loss (1.03/100 person-years). HBsAg loss was significantly associated with a 0.5–1 log10 (treatment-naïve: adjusted hazard ratio [aHR] 8.06, 95% confidence interval [CI] 1.29–50.40; treatment-experienced: aHR 4.34, 95% CI 1.40–13.47) and >1 log10 qHBsAg decrease (treatment-naïve: aHR 9.19, 95% CI 1.47–57.65; treatment-experienced: aHR 8.02, 95% CI 1.76–36.57) compared with qHBsAg not reduced. HBsAg loss was significantly associated with lower baseline qHBsAg in treatment-experienced cohort, while such difference was not significant in treatment-naïve cohort.

**DISCUSSION:**

A rapid decline of qHBsAg in 12 months during NAs therapy, as opposed to merely maintaining a low level of qHBsAg, was associated with HBsAg loss.

## INTRODUCTION

Chronic hepatitis B (CHB) infection is a worldwide health problem that is estimated to affect 257.5 million persons (1). Active replication of hepatitis B virus (HBV) is the key driver of hepatic inflammation and disease progression. Current treatments of CHB are pegylated interferon (Peg-IFN) and nucleos(t)ide analogs (NAs) (2). As the first-line treatment, NAs act to suppress viral DNA synthesis through inhibition of reverse transcriptase (3). The aim of treatment of CHB was to permanently suppress viral replication, as evidenced by loss of serum hepatitis B surface antigen (HBsAg) (functional cure), undetectable serum HBV DNA, and HBsAg seroconversion (4). The treatment goal was to reduce the risk of disease progression and to improve quality of life.

HBsAg is the glycosylated envelope protein of mature hepatitis B virions and is an important prognostic indicator of response to antiviral therapy. HBsAg can be produced by closed circular DNA or from HBV DNA integrated into the hepatocyte nucleus. Quantitation of HBsAg (qHBsAg) is increasingly used as a diagnostic and prognostic tool and can be used to guide treatment decisions in patients with CHB (5). For example, high level of HBsAg is used to identify poor responders to Peg-IFN (5). Furthermore, based on longitudinal qHBsAg data, Fan et al (6) developed a new model which accurately predicts HBsAg clearance, provides reliable estimates of functional HBV cure. HBsAg loss is the optimal outcome for patients with CHB, but this rarely occurs with currently approved therapies. Patients who achieve HBsAg loss during NAs treatment showed better clinical outcomes overall than those who unable to clear HBsAg (7). However, treatment with NAs rarely leads to complete viral eradication and HBsAg loss. Often, patients required prolonged or lifelong therapy to prevent viral relapse (4,8).

An early decline in HBsAg is predictive of HBsAg loss in patients treated with Peg-IFN (9) and is thought to predict the treatment response to NAs, although the optimal time for testing has not been determined and ranges from 3 to 12 months (10,11). A decline in HBsAg of >1 log10 IU/mL after 1 year of treatment or an early decline of >1 log10 IU/mL after 12–48 weeks in HBV e antigen (HBeAg)-positive patients has been reported as predictive for HBsAg loss (12,13). A case-control study in patients who underwent spontaneous seroclearance of HBsAg found that an HBsAg level <200 IU/mL combined with a ≥1 log10 IU/mL decrease in HBsAg was predictive of HBsAg loss in the next 1–3 years (positive predictive value 97% and 100%, respectively) (14). Available evidence suggests that a decline in HBsAg might be predictive of HBsAg loss in the HBeAg-positive patients (5). However, in patients during NAs treatment, a decline in HBsAg within one year in predicting the prognosis remained unclear.

The aim of this study was to investigate associations between qHBsAg reduction and HBsAg loss in patients with CHB who under NAs treatment. This study was conducted in China and contributes to the body of knowledge supporting the use of HBsAg as a prognostic indicator in patients with NAs treatment.

## METHODS

### Data collection

This was a cohort study using electronic medical records from Ruijin Hospital, Shanghai Jiao Tong University School of Medicine, a tertiary general hospital in China that provides high-quality care for patients, including those with CHB. The data consisted of deidentified patient records with unique identifiers linking patient information including demographic details, clinical, prescription, and pathological information. The study was approved by the Human Ethics Committee, Ruijin Hospital, Shanghai Jiao Tong University School of Medicine. Consistent with the Chinese Health Insurance Portability and Accountability Act, patient consent was not required for this noninterventional, retrospective study using deidentified electronic data.

### Study population

The base study population included all patients with at least one diagnosis of CHB who had received NAs treatment between January 01, 2012, and December 31, 2020, in the study hospital. The study start date corresponded to the period when HBsAg quantification commenced at the hospital.

CHB diagnoses were identified from medical records using *International Classification of Diseases 10th Revision* (*ICD-10*) code B18.1 plus free-text diagnoses. All free-text diagnoses of CHB were reviewed and validated by infectious disease specialists at the hospital. NAs treatment was identified from prescription records and included lamivudine, adefovir, entecavir, telbivudine, tenofovir alafenamide fumarate and tenofovir administered as mono- or combined treatments.

The index date was 12 months after the first identified date of NAs treatment. The baseline period is defined as the 3-month period before the first identified date of NAs treatment, with the date closest to the identified date. The 12-month period between the first identified date of NAs treatment and the index date was the observation period for HBsAg reduction. Patients were followed up from the index date until the discontinuation of NAs treatment (identified by gap more than 1 year between 2 prescriptions), end of study period, or the last visit showed in the medical record, whichever occurred first.

The study population include all patients aged 18 years or older with gender information and baseline qHBsAg ≥0.05 IU/mL (corresponding to a positive HBsAg measurement), which is the most recent qHBsAg test before the first identified NAs treatment, and with at least one qHBsAg level taken during 12-month NAs treatment. Patients were excluded if they had coinfection with hepatitis A, C, D, E virus or human immunodeficiency virus, a diagnosis of hepatocellular carcinoma or any other malignancy before the index date, if they had received NAs for <12 months, if they were receiving interferon treatment concurrently with NAs, or if they achieved HBsAg loss (qHBsAg <0.05 IU/mL or HBsAg negative) during the observation period were excluded. Study populations were grouped into 2 cohorts: treatment-experienced cohort included patients with undetectable HBV DNA, which indicated previous NAs treatment, and treatment-naïve cohort included the rest treatment-naïve patients.

### Exposure

Exposure includes the baseline qHBsAg level and the change in qHBsAg. The change in qHBsAg was defined as qHBsAg level trend within 12-month NAs treatment. Multiple measurements were compared with the base qHBsAg level (the most recent qHBsAg test before the first identified NAs treatment). If there were more reduction measurements, the reduced one closest to the index date was used for reduced trend. If there were more nonreduction ones, the nonreduced one closest to the index date was used.

### Study end point

The primary end point was HBsAg loss, defined as a serum HBsAg <0.05 IU/mL or a negative report of HBsAg at least 14 days after the 12-month assessment of qHBsAg level when change in qHBsAg was determined.

### Laboratory tests

HBsAg was quantified by Abbott Architect HBsAg Reagent Kit (Abbott Ireland Diagnostics Division, Sligo, Ireland). Serum HBeAg was measured using a commercial enzyme immunoassay kit (AXSYM System; Abbott, Wiesbaden, Germany). Serum HBV DNA quantification was determined using Applied Biosystems real-time polymerase chain reaction system Prism 7500 (Applied Biosystems, Waltham, MA; with a lower limit of 500 IU/mL) or Roche COBAS HBV Amplicor Monitor assay (Roche Diagnostics, Basel, Switzerland; with a lower limit of 20 IU/mL).

### Statistical analysis

Continuous variables were described by median and interquartile range (IQR). Categorical variables were presented using counts and percentages, as appropriate. Pearson correlation coefficient was used. In the study cohorts, the incidence rates of HBsAg loss were estimated. The change in qHBsAg after 12 months of NAs treatment was categorized as “no reduced” (including increasing, not reducing, or reduced by < 0.5 log10 IU/mL), “reduced by 0.5–1 log10 IU/mL” or “reduced by > 1 log10 IU/mL”. Univariable and multivariable Cox proportional hazards models were conducted to assess associations between baseline qHBsAg level, as well as change in qHBsAg after 12 months and HBsAg loss during the cohort follow-up period. In the models, potential confounders, including the following parameters change in HBeAg status at baseline, baseline laboratory results for alanine aminotransferase (ALT) level, baseline diagnoses of hypertension or diabetes, cirrhosis, age, and sex, were adjusted. For laboratory results, if there were multiple measurements at baseline, the one closest to the first identified NAs treatment was used.

Statistical analyses were conducted using SAS Version 9.4 (SAS Institute, Cary, NC).

## RESULTS

### Demographic and clinical characteristics of the study cohorts

**Figure 1.**
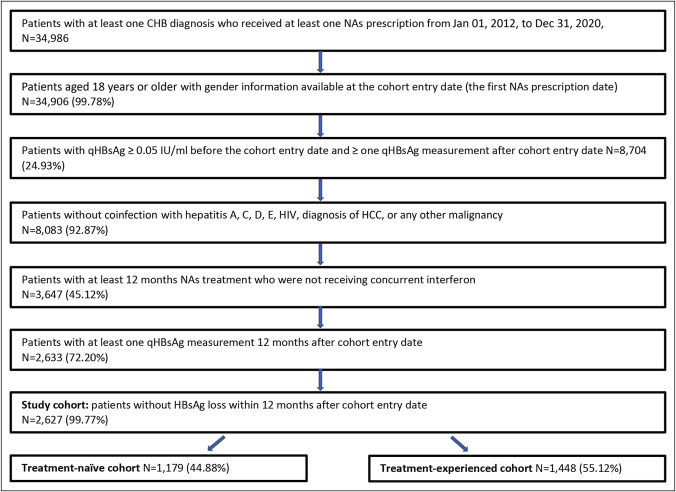
Patient selection. CHB, chronic hepatitis B; HBeAg, hepatitis B e antigen; HCC, hepatocellular carcinoma; NAs, nucleos(t)ide analogs; qHBsAg, quantitative hepatitis B surface antigen level.

In overall, 2,627 patients were identified among 34,986 patients with CHB who received NAs treatment from January 01, 2012, to December 31, 2020, at Ruijin Hospital, and treatment-naïve cohort and treatment-experienced cohort included 1,179 and 1,448 patients, respectively (Figure 1). In the treatment-naïve cohort, the median age of patients was 36 years (IQR 30–46), and 770 (65.31%) were male (Table 1). At baseline, 81.09% patients had qHBsAg quantification of over 1,000 IU/mL, 64.89% patients were HBeAg positive, and 40.80% had ALT level over 2 times the upper limit of normal. Cirrhosis was present in 59 patients (5.00%), nonalcoholic fatty liver disease in 46 (3.90%), and hypertension and diabetes were present in 23 (1.95%) and 12 patients (1.02%), respectively. Patients were treated predominantly with entecavir (58.44%) or tenofovir (21.29%). In the treatment-experienced cohort, patients were elder than patients in the treatment-naïve cohort with median age of 42 years (34–53.2), and 977 (67.47%) were male. At baseline, compared with treatment-naïve patients, fewer patients in treatment-experienced cohort got HBsAg quantification of over 1,000 IU/mL, had HBeAg positive condition, and had ALT level over 2 upper limit of normal. Other clinical characteristics of comorbidities were similar. Patients were treated predominantly with entecavir (50.41%) or lamivudine (13.33%).

**Table 1.** Demographic and clinical characteristics of study population

Variable	Treatment-naïve cohort N = 1,179	Treatment-experienced cohort N = 1,448
Age at cohort entry date (yr)		
Median (IQR)	36 (30–46)	42 (34–53.2)
Range, n (%)	19–79	18–82
18–30	309 (26.21)	213 (14.71)
31–40	442 (37.49)	448 (30.94)
41–50	194 (16.45)	336 (23.2)
51–60	150 (12.72)	287 (19.82)
>60	84 (7.12)	164 (11.33)
Gender, n (%)		
Male	770 (65.31)	977 (67.47)
Female	409 (34.69)	471 (32.53)
HBeAg, n (%)		
Negative	367 (31.13)	787 (54.35)
Positive	765 (64.89)	590 (40.75)
Missing	47 (3.99)	71 (4.90)
qHBsAg, IU/mL, n (%)		
<100	64 (5.4%)	158 (10.9%)
101-1,000	159 (13.5%)	376 (26.0%)
1,001-10000	524 (44.4%)	791 (54.6%)
>10,000	432 (36.6%)	123 (8.5%)
ALT, IU/L n (%)		
≤80^a^	628 (53.27)	1,384 (95.58)
>80	481 (40.80)	44 (3.04)
Missing	70 (5.94)	20 (1.38)
Cirrhosis, n (%)	59 (5.00)	142 (9.80)
MASLD, n (%)	46 (3.90)	26 (1.79)
Hypertension, n (%)	23 (1.95)	43 (2.97)
Diabetes, n (%)	12 (1.02)	23 (1.59)
NAs treatment at cohort entry date, n (%)		
Entecavir	689 (58.44)	730 (50.41)
Tenofovir	251 (21.29)	191 (13.19)
Telbivudine	113 (9.58)	158 (10.91)
Lamivudine	48 (4.07)	193 (13.33)
Adefovir	69 (5.85)	170 (11.74)
Tenofovir alafenamide fumarate	9 (0.76)	6 (0.41)

ALT, alanine aminotransferase; HBeAg, hepatitis B e antigen; HBV, hepatitis B virus; IQR, interquartile range; MASLD, metabolic dysfunction-associated steatotic liver disease; NAs, nucleos(t)ide analogs; qHBsAg, quantitative hepatitis B surface antigen level.

a Twice the upper limit of normal.

### Baseline qHBsAg showed positive correlation with qHBsAg reduction in 12-month NAs treatment

Considering baseline qHBsAg as an important predictor of HBsAg loss, the relationship between qHBsAg and reduction in qHBsAg level was further explored. Owing to the variability in baseline qHBsAg levels, using the absolute reduction in qHBsAg values to represent the magnitude of decline would be biased. Thus, we analyzed the correlation between the baseline qHBsAg and the qHBsAg reduction in log value. In the treatment-naïve cohort, a significant positive correlation was observed between baseline qHBsAg level and reduction in qHBsAg (*r* = 0.40). Among patients with baseline qHBsAg levels less than 10,000 IU/mL, 11.91% demonstrated a reduction of ≥ 0.5 log10 IU/mL. By contrast, among those with baseline qHBsAg levels greater than 10,000 IU/mL, 51.15% achieved a reduction of ≥ 0.5 log10 IU/mL. A weaker positive correlation was noted in the treatment-experienced cohort (*r* = 0.13), where 14.63% of patients with high baseline qHBsAg levels (>10,000 IU/mL) exhibited a reduction of ≥ 0.5 log10 IU/mL (Table S1, http://links.lww.com/CTG/B268).

### Significant qHBsAg reduction in 12-month NAs treatment was positively correlative with HBsAg loss

In treatment-naïve cohort, 6 of 1,179 patients experienced HBsAg loss during the 12-month observation period, and thus were excluded in the subsequent Cox regression analysis. There were 9 patients (0.77%) who achieved HBsAg loss during the follow-up, giving an incidence rate of 0.51 per 100 person-years (Table 2). The median (IQR) time to HBsAg loss was 910 (352–1,323) days. The estimated incidence rate of HBsAg loss was 1.26 per 100 person-years in patients with >1 log10 IU/mL qHBsAg decrease, 1.31 per 100 person-years in patients with 0.5–1 log10 IU/mL qHBsAg decrease, and 0.23 per 100 person-years in patients with no reduced qHBsAg.

**Table 2.** Univariate and multivariate analysis of qHBsAg reduction and baseline qHBsAg associated with HBsAg loss (Cox proportional hazards regression model) among the treatment-naïve cohort

Risk factor	No. of patients	No. with HBsAg loss	Follow-up (person-years)	Incidence rate	Crude HR (95% CI)	Adjusted HR^a^ (95% CI)
qHBsAg compared with baseline						
No reduced	866	3	1,312.44	0.23	Ref	Ref
Reduced 0.5–1 log	158	3	229.41	1.31	5.65 (1.14–28.01)*	8.06 (1.29–50.4)*
Reduced >1 log	149	3	237.77	1.26	5.35 (1.08–26.52)*	9.19 (1.47–57.65)*
qHBsAg at baseline						
<100	61	1	69.16	1.45	Ref	Ref
101–1,000	159	3	261.75	1.15	0.63 (0.06–6.19)	0.64 (0.06–6.92)
1,001–10,000	523	2	805.39	0.25	0.15 (0.01–1.62)	0.14 (0.01–2.03)
>10,000	430	3	643.31	0.47	0.28 (0.03–2.71)	0.37 (0.02–6.9)

ALT, alanine aminotransferase; CI, confidence interval; HBeAg, hepatitis B e antigen; HBV, hepatitis B virus; HR, hazard ratio; qHBsAg, quantitative hepatitis B surface antigen; ref, reference.

*Statistically significant at p < 0.05.

a Adjusted for age, gender, HBeAg status, ALT level, cirrhosis, and comorbidity (diabetes and hypertension) at baseline.

**Figure 2.**
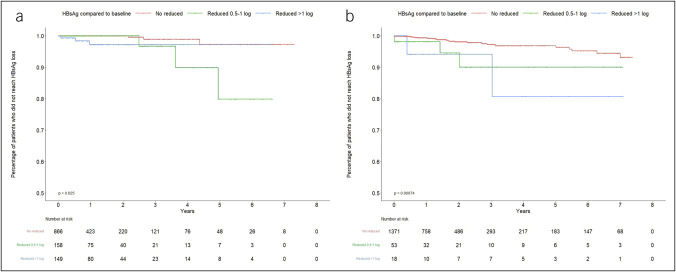
Cumulative incidence of HBsAg loss stratified by decline in qHBsAg levels. Cumulative incidence of HBsAg loss stratified by decline in qHBsAg levels among the treatment-naïve cohort (**a**) and treatment-experienced cohort (**b**). HBsAg, hepatitis B surface antigen; qHBsAg, quantitative hepatitis B surface antigen level.

The rate of HBsAg loss was statistically significantly associated with qHBsAg decrease (Table 2, Figure 2a). There was a relatively higher rate of HBsAg loss in patients with >1 log10 IU/mL decrease in qHBsAg (adjusted hazard ratio (aHR) 9.19, 95% CI 1.47–57.65) and in patients with 0.5–1 log10 IU/mL decrease in qHBsAg (aHR 8.06, 95% CI 1.29–50.40), compared with patient had no reduced qHBsAg.

In the treatment-experienced cohort, 6 of 1,448 patients experienced HBsAg loss during the 12-month observation period and were also excluded. The incidence rate of HBsAg loss was 1.03 per 100 person-years during the follow-up period (Table 3). The median (IQR) time to HBsAg loss was 601.5 (240.8–1,093) days. The estimated incidence rate of HBsAg loss was the highest in patients with the most log decreases in qHBsAg after 12 months of enrollment: 4.63 per 100 person-years in patients with >1 log10 IU/mL qHBsAg decrease, vs 3.73 per 100 person-years in patients with 0.5–1 log10 IU/mL qHBsAg decrease, and 0.87 per 100 person-years in patients with no reduced qHBsAg over time. The rate of HBsAg loss was statistically significantly associated with qHBsAg decrease (>1 log10 IU/mL decrease: aHR 8.02, 95% CI 1.76–36.57; 0.5–1 log10 IU/mL decrease: aHR 4.34, 95% CI 1.40–13.47) (Table 3, Figure 2b).

**Table 3.** Univariate and multivariate analysis of qHBsAg reduction and baseline qHBsAg associated with HBsAg loss (Cox proportional hazards regression model) among the treatment-experienced cohort

Risk factor	No. of patients	No. with HBsAg loss	Follow-up (person-years)	Incidence rate	Crude HR (95% CI)	Adjusted HR^a^ (95% CI)
qHBsAg compared to baseline						
No reduced	1,371	24	2,760.59	0.87	Ref	Ref
Reduced 0.5–1 log	53	4	107.18	3.73	4.26 (1.48–12.28)*	4.34 (1.4–13.47)*
Reduced >1 log	18	2	43.23	4.63	5.87 (1.38–24.93)*	8.02 (1.76–36.57)*
qHBsAg at baseline						
<100	154	16	245.48	6.52	Ref	Ref
101–1,000	376	6	821.81	0.73	0.10 (0.04–0.27)*	0.08 (0.03–0.23)*
1,001–10,000	789	7	1,606.27	0.44	0.06 (0.03–0.15)*	0.06 (0.02–0.15)*
>10,000	123	1	237.44	0.42	0.06 (0.01–0.48)*	0.05 (0.01–0.43)*

ALT, alanine aminotransferase; CI, confidence interval; HBeAg, hepatitis B e antigen; HBV, hepatitis B virus; HR, hazard ratio; qHBsAg, quantitative hepatitis B surface antigen; ref, reference.

*Statistically significant at p < 0.05.

a Adjusted for age, gender, HBeAg status, ALT level, cirrhosis, and comorbidity (diabetes and hypertension) at baseline.

### Baseline qHBsAg level was negatively correlative with HBsAg loss

**Figure 3.**
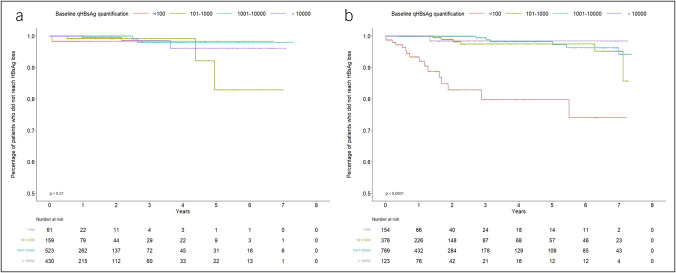
Cumulative incidences of HBsAg loss stratified by baseline qHBsAg levels. Cumulative incidence of HBsAg loss stratified by baseline qHBsAg levels in among the treatment-naïve cohort (**a**) and treatment-experienced cohort (**b**). HBsAg, hepatitis B surface antigen; qHBsAg, quantitative hepatitis B surface antigen level.

In the treatment-naïve cohort, the estimated incidence rate of HBsAg loss was the highest in patients with the lowest baseline qHBsAg level: 1.45 per 100 person-years in patients with <100 IU/mL, vs 1.15 per 100 person-years in patients with 101–1,000 IU/mL, 0.25 per 100 person-years in patients with 1,001–10,000 IU/mL and 0.47 per 100 person-years in patients with over 10,000 IU/mL. Compared with patients with lowest baseline level, patients in other groups had lower rate of HBsAg loss while such difference was not statistically significant (Table 2, Figure 3a).

In the treatment-experienced cohort, patients with the lowest baseline qHBsAg level also had the highest incidence rate of HBsAg loss of 6.52 per 100 person-years, vs 0.73 per 100 person-years in patients with 101–1,000 IU/mL, 0.44 per 100 person-years in patients with 1,001–10,000 IU/mL, and 0.42 per 100 person-years in patients with over 10,000 IU/mL. The rate of HBsAg loss was statistically significantly associated with baseline qHBsAg level. There was a lower rate of HBsAg loss in patients with 101–1,000 IU/mL (aHR 0.08, 95% CI 0.03–0.23), in patients with 1,001–10,000 IU/mL (aHR 0.06, 95% CI 0.02–0.15), and in patients with over 10,000 IU/mL (aHR 0.05, 95% CI 0.01–0.43), compared with patients with <100 IU/mL (Table 3, Figure 3b).

### Association between other factors and HBsAg loss

Association between other factors, including age, gender, baseline HBeAg status, ALT level, cirrhosis and comorbidity of diabetes and hypertension, and HBsAg loss were presented in Tables S2 (http://links.lww.com/CTG/B269) and S3 (http://links.lww.com/CTG/B270). In both cohorts, no significant association was found between these characters and HBsAg loss, except for comorbidity with diabetes. In the treatment-experienced cohort, a higher rate of HBsAg loss was observed in patients with diabetes in univariable model, and the difference remained significant after adjusting for other variables (aHR 4.72, 95% CI 1.25–17.85).

## DISCUSSION

In this study, the low rate of HBsAg loss is observed in both treatment-naïve and treatment-experienced cohort, while patients in treatment-experienced cohort had a relatively higher incidence rate of 1.03 per 100 person-years compared with that in treatment-naïve cohort (0.51 per 100 person-years). Globally, HBsAg loss occurs infrequently in patients with CHB, and the result from this study consists with global situation. The rate is generally in line with the rates of 0.82 per 100 person-years reported in a systematic review and meta-analysis by Yeo and colleagues (15) and higher than 0.33 per 100 person-years reported in Korean patients receiving lamivudine or entecavir treatment (16). Our finding of low HBsAg loss incidence expands on published studies that were limited by smaller sample size, shorter follow-up time, or older NAs with significant viral resistance.

Serum HBsAg level was reported as a good predictor of long-term HBsAg loss in patients with CHB, and a low baseline level is typically considered to predict the HBsAg loss. Our study yielded consistent findings that patients with lower baseline HBsAg levels exhibited a higher rate of HBsAg loss when compared with those in groups with higher levels, a pattern particularly pronounced among patients with prior treatment experience. Concurrently, we observed that patients experienced a spectrum of reductions in their baseline HBsAg levels. In this study, we further assessed the correlation between HBsAg reduction and HBsAg loss, discovering a positive association between the decrease in qHBsAg levels and the occurrence of HBsAg loss. The incidence rate of HBsAg loss increased proportionally with the observed decrease in qHBsAg with 12-month NAs treatment, and HBsAg loss was statistically significant for a 0.5–1 log10 IU/mL decline and >1 log10 IU/mL decline compared with no decrease in both cohorts. This finding indicated that NAs treatment-induced quantitative declining of HBsAg predicts potential cure of CHB (HBsAg loss), no matter the patient is NAs treatment-naïve or has been previously treated. From the results, the decline in HBsAg had a more significant effect on HBsAg loss in treatment-naïve cohort than in treatment-experienced cohort, but the limited number of patients with HBsAg loss yielded a wide confidence interval. Our findings among patients with NAs treatment showed consistent trend with other studies that had proposed that a decline in HBsAg increased the treatment response to NAs in patients with CHB (10,11,17–19). Compared with these previous studies which mostly investigated treatment-naïve patients with CHB or did not clarify the treatment status, our study assessed such association in treatment-naïve and treatment-experienced patients separately, thus further informing that decline in qHBsAg could predict HBsAg loss at different courses of CHB patients with NAs treatment. Our study suggested not only the decline at 12 months with initial NAs but also with the previously treated NAs seemed to be a time point with clinical relevance in predicting response. Based on the results, evaluation of on-treatment HBsAg changes might exert potential clinical utility to monitor therapeutic response in management of patients with CHB receiving NAs treatment.

Strengths of our study include the availability of a large patient cohort and extended follow-up period that allowed us to detect HBsAg loss that occur infrequently in patients with CHB. Potential study limitations include unavailability of the data of the start of NAs treatment for the treatment-experienced patients. The qHBsAg reduction was observed from the first identified NAs treatment but not necessarily from the start of NAs treatment; thus, the reduction evaluated may occurred in any stage of NAs treatment. Besides, as a retrospective observational research based on real-world practice data, residual confounders are a concern. Specialized clinical tests such as HBV genotype, liver fibrosis stage, and lifestyle factors such as smoking habits, alcohol use, and diet were not captured in the hospital database.

It provided a scientific basis for developing personalized treatment plans. In view of the clinical situation of different patients, health care decision-makers should incorporate this factor into the evaluation system to optimize the treatment and improve the quality of life and clinical outcome of patients.

A significant and rapid decline in qHBsAg in 12-month NAs treatment, as opposed to merely maintaining a very low level of qHBsAg, was statistically significantly associated with eventual loss of HBsAg in Chinese patients with CHB. This association was observed in both patients who were new to NAs treatment and those with prior NAs treatment experience. The rate of HBsAg loss was marginally higher among NAs treatment-experienced patients compared with those who were treatment-naïve.

## CONFLICTS OF INTEREST

**Guarantor of the article:** Wei Cai, MD, PhD.

**Specific author contributions:** T.Z.: formal analysis, funding acquisition, manuscript writing. M.S.: supervision, formal analysis, manuscript writing. F.L.: supervision, manuscript writing. Si Jia: investigation, conceptualization, methodology. J.T.: investigation, data curation. Y.D.: data curation, supervision, writing review & editing. H.Q.: formal analysis, conceptualization, writing review & editing. W.C.: conceptualization, supervision, funding acquisition, manuscript writing.

**Financial support:** This study was funded by the National Natural Science Foundation of China (82100646, 81470867), the National Thirteenth Five Year Plan Major Special Project (2017ZX09304016).

**Potential competing interests:** H.Q., M.S., and S.D. are full-time employees of Janssen Research & Development. H.Q., M.S. holds stocks of Johnson & Johnson. No other conflicts of interests are declared by the authors.

**Institutional Review Board statement:** The study was meticulously aligned with the ethical tenets stipulated in the Declaration of Helsinki Istanbul, and its implementation was sanctioned by the Human Ethics Committee of Ruijin Hospital (Reference No. 2021-150). Considering the retrospective nature of the study, the Committee granted the exemption of written informed consent.

**Study Highlights**

**WHAT IS KNOWN**

✓ Loss of hepatitis B surface antigen (HBsAg) is the pivotal component of functional cure in patients suffering from chronic hepatitis B.
✓ Low levels of HBsAg, or an early rapid decline in HBsAg after medication treatment, could be predictive of HBsAg loss in patients treated with pegylated interferon or nucleos(t)ide analogs.

**WHAT IS NEW HERE**

✓ The significant and rapid decline of quantitative HBsAg during 12-month nucleos(t)ide analogs therapy serves as an important biomarker indicating the HBsAg loss, in both treatment-naïve and treatment-experienced patients with chronic hepatitis B.

## Supplementary Material
