# Supplementary material for: Quantitative Change of Hepatitis B Surface Antigen Leading to Final Hepatitis B Surface Antigen Loss in Patients with Chronic Hepatitis B Receiving Nucleos(t)ide Analogs in China
Source: Clin Transl Gastroenterol. 2025 Jan 16;16(4):e00820. doi: 10.14309/ctg.0000000000000820 (PMC12020684; doi:10.14309/ctg.0000000000000820)
Supplement: Supplementary file 1 [file ct9-16-e00820-s001.docx]

**Table S1. Association between baseline qHBsAg and qHBsAg reduction in 12-month NAs treatment.**

|  | **Treatment-naïve cohort** | | | **Treatment-experienced cohort** | | |
| --- | --- | --- | --- | --- | --- | --- |
| Baseline qHBsAg, IU/ml, n (%) | No reduced | Reduced 0.5–1 log | Reduced >1 log | No reduced | Reduced 0.5–1 log | Reduced >1 log |
| <100 | 52 (81.25) | 5 (7.81) | 7 (10.94) | 145 (91.77) | 8 (5.06) | 5 (3.16) |
| 101-1000 | 147 (92.45) | 6 (3.77) | 6 (3.77) | 349 (92.82) | 25 (6.65) | 2 (0.53) |
| 1001-10000 | 459 (87.60) | 50 (9.54) | 15 (2.86) | 776 (98.10) | 10 (1.26) | 5 (0.63) |
| >10000 | 211 (48.84) | 97 (22.45) | 124 (28.70) | 105 (85.37) | 10 (8.13) | 8 (6.50) |
|  |  |  |  |  |  |  |

qHBsAg, quantitative hepatitis B surface antigen; ALT, alanine aminotransferase; CI, confidence interval; HBeAg, hepatitis B e antigen; HBV, hepatitis B virus; HR, hazard ratio; ref, reference

**^a^** adjusted for age, gender, HBeAg status, ALT level, cirrhosis and comorbidity (diabetes and hypertension) at baseline.
