# Supplementary material for: Quantitative Change of Hepatitis B Surface Antigen Leading to Final Hepatitis B Surface Antigen Loss in Patients with Chronic Hepatitis B Receiving Nucleos(t)ide Analogs in China
Source: Clin Transl Gastroenterol. 2025 Jan 16;16(4):e00820. doi: 10.14309/ctg.0000000000000820 (PMC12020684; doi:10.14309/ctg.0000000000000820)
Supplement: Supplementary file 2 [file ct9-16-e00820-s002.docx]

**Table S2. Univariate and multivariate analysis of other factors associated with HBsAg loss (Cox proportional hazards regression model) among the treatment-naïve cohort.**

| **Risk factor** | **No. of patients** | **No. with HBsAg loss** | **Follow-up (person-years)** | **Incidence rate** | **Crude HR**  **(95% CI)** |  |
| --- | --- | --- | --- | --- | --- | --- |
| Baseline HBeAg status |  |  |  |  |  |  |
| Negative | 366 | 5 | 524.74 | 0.95 | ref |  |
| Positive | 761 | 4 | 1197.89 | 0.33 | 0.36(0.10-1.34) |  |
| Missing* | 46 | 0 | 56.99 | 0.00 |  |  |
| Age |  |  |  |  |  |  |
| 18-30 | 307 | 3 | 387.25 | 0.77 | ref |  |
| 31-40 | 442 | 3 | 671.23 | 0.45 | 0.53(0.11-2.63) |  |
| 41-50 | 194 | 1 | 292.92 | 0.34 | 0.36(0.04-3.54) |  |
| 51-60 | 147 | 2 | 269.46 | 0.74 | 0.73(0.12-4.48) |  |
| >60 | 83 | 0 | 158.75 | 0.00 | 0(0-Inf) |  |
| Gender |  |  |  |  |  |  |
| Male | 768 | 5 | 1193.65 | 0.42 | ref |  |
| Female | 405 | 4 | 585.97 | 0.68 | 1.66(0.44-6.17) |  |
| ALT level |  |  |  |  |  |  |
| ≤80 | 628 | 4 | 898.52 | 0.45 | ref |  |
| >80 | 476 | 5 | 795.54 | 0.63 | 1.33（0.36-4.98） |  |
| Missing* | 69 | 0 | 85.56 | 0.00 |  |  |
| Diabetes |  |  |  |  |  |  |
| No | 1161 | 9 | 1764.86 | 0.51 | ref |  |
| Yes | 12 | 0 | 14.75 | 0.00 | 0(0-Inf) |  |
| Hypertension |  |  |  |  |  |  |
| No | 1151 | 9 | 1740.98 | 0.52 | ref |  |
| Yes | 22 | 0 | 38.36 | 0.00 | 0(0-Inf) |  |
| Cirrhosis |  |  |  |  |  |  |
| No | 1115 | 8 | 1692.30 | 0.47 | ref |  |
| Yes | 58 | 1 | 87.31 | 1.15 | 2.43(0.30-19.59) |  |
|  |  |  |  |  |  |  |

ALT, alanine aminotransferase; CI, confidence interval; HBeAg, hepatitis B e antigen; HR, hazard ratio; ref, reference

* Missing records were not included in the Cox regression model.
