# Supplementary material for: Quantitative Change of Hepatitis B Surface Antigen Leading to Final Hepatitis B Surface Antigen Loss in Patients with Chronic Hepatitis B Receiving Nucleos(t)ide Analogs in China
Source: Clin Transl Gastroenterol. 2025 Jan 16;16(4):e00820. doi: 10.14309/ctg.0000000000000820 (PMC12020684; doi:10.14309/ctg.0000000000000820)
Supplement: Supplementary file 3 [file ct9-16-e00820-s003.docx]

**Table S3. Univariate and multivariate analysis of other factors associated with HBsAg loss (Cox proportional hazards regression model) among the treatment-experienced cohort.**

| **Risk factor** | **No. of patients** | **No. with HBsAg loss** | **Follow-up (person-years)** | **Incidence rate** | **Crude HR**  **(95% CI)** |  |
| --- | --- | --- | --- | --- | --- | --- |
| Baseline HBeAg status |  |  |  |  |  |  |
| Negative | 783 | 18 | 1514.84 | 1.19 | ref |  |
| Positive | 588 | 12 | 1248.32 | 0.96 | 0.8(0.38-1.66) |  |
| Missing* | 71 | 0 | 147.84 | 0.00 |  |  |
| Age |  |  |  |  |  |  |
| 18-30 | 212 | 2 | 304.26 | 0.66 | ref |  |
| 31-40 | 448 | 7 | 879.92 | 0.80 | 1.23(0.26-5.96) |  |
| 41-50 | 336 | 6 | 662.49 | 0.91 | 1.35(0.27-6.71) |  |
| 51-60 | 283 | 6 | 666.53 | 0.90 | 1.36(0.27-6.78) |  |
| >60 | 163 | 9 | 397.81 | 2.26 | 3.32(0.71-15.54) |  |
| Gender |  |  |  |  |  |  |
| Male | 974 | 20 | 1914.15 | 1.04 | ref |  |
| Female | 468 | 10 | 996.85 | 1.00 | 0.93(0.44-1.99) |  |
| ALT level |  |  |  |  |  |  |
| ≤80 | 1379 | 30 | 2783.72 | 1.08 | ref |  |
| >80 | 43 | 0 | 83.50 | 0.00 | 0(0-Inf) |  |
| Missing* | 20 | 0 | 43.78 | 0.00 |  |  |
| Diabetes |  |  |  |  |  |  |
| No | 1419 | 27 | 2860.13 | 0.94 | ref |  |
| Yes | 23 | 3 | 50.66 | 5.92 | 6.14(1.86-20.3) |  |
| Hypertension |  |  |  |  |  |  |
| No | 1399 | 27 | 2806.13 | 0.96 | ref |  |
| Yes | 43 | 3 | 104.87 | 2.86 | 3.1(0.94-10.25) |  |
| Cirrhosis |  |  |  |  |  |  |
| No | 1301 | 28 | 2653.31 | 1.06 | ref |  |
| Yes | 141 | 2 | 257.68 | 0.78 | 0.73(0.17-3.08) |  |
|  |  |  |  |  |  |  |

ALT, alanine aminotransferase; CI, confidence interval; HBeAg, hepatitis B e antigen; HR, hazard ratio; ref, reference

*, Missing records were not included in the Cox regression model.
